# Revisiting symptom dimensions in schizophrenia with exploratory graph analysis

**DOI:** 10.1038/s41537-026-00799-y

**Published:** 2026-09-07

**Authors:** Susan Illing, Stefan Leucht

**Affiliations:** Section for Evidence-Based Medicine in Psychiatry and Psychotherapy, Department of Psychiatry and Psychotherapy, School of Medicine and Health, Technical University of Munich, Munich, Germany

**Keywords:** Schizophrenia, Psychiatric disorders

## Abstract

The Positive and Negative Syndrome Scale (PANSS) groups symptoms into three categories, but factor analyses suggest five dimensions with superior psychometric properties. Exploratory graph analysis (EGA) offers a novel network-based approach that provides additional information such as connection of domains, bridge symptoms, and centrality measures. We applied EGA to PANSS data from 1213 participants with schizophrenia at baseline and after six weeks of treatment. We identified symptom clusters with community detection algorithms, item stability with bootstrapping, and the connecting role of individual items with bridge centrality metrics. EGA consistently revealed five communities: positive symptoms, negative symptoms, cognitive disorganization, depression/anxiety, and hostility/excitement. The latter was less stable and its symptom P6 (suspiciousness) moved to the positive symptom domain at endpoint. Other item assignments were stable, except for items P5 (grandiosity), and G8 (uncooperativeness). P4 (excitement), P6, G8, and G15 (preoccupation) emerged as key bridge symptoms. Overall, the replication of five factors with a different method underlines their clinical importance.

## Background

The Positive and Negative Syndrome Scale (PANSS)^1^ is the most commonly used scale for measuring symptoms of schizophrenia. It was conceptualized as a categorical system covering positive, negative, and general symptoms, which in part reflected the Type 1 (predominantly positive symptoms, acute presentation) and Type 2 (predominantly negative symptoms, chronic course) schizophrenia subtypes by Crow^2^. Nevertheless, subsequent factor analyses questioned this categorical concept making the PANSS structure a subject of ongoing debate^3,4^. Roithmeier et al. systematically reviewed 95 PANSS factor models and found that five-factor solutions were the most frequent and demonstrated the best fit indices^5^. Among these, the consensus model by Wallwork et al. is particularly notable, as it synthesized 29 published variations of five-factor structures^6^. This model includes 20 of the original 30 PANSS items, grouped into the factors positive, negative, disorganized/concrete, excited, and depressed. Similarly, the modified approach by van der Gaag et al. produced robust results^7^. Combining exploratory factor analysis (EFA) and confirmatory factor analysis (CFA) with 10-fold cross-validation, their method allowed items to load on more than one factor. These secondary loadings were argued to better capture the highly interrelated symptoms of schizophrenia, resulting in factors defined by both primary and secondary loadings. In contrast, the Marder model^8^, frequently used in pharmacological studies, demonstrated insufficient psychometric properties and is therefore not recommended for use^5^.

Traditionally, such models have been derived from classical factor-analytic methods and principal component analyses. As an alternative, only few recent studies applied exploratory graph analysis (EGA)^9–12^, a network modeling approach that identifies clusters (communities) of highly correlated items^13^. EGA allows for visual inspection of the network structure and provides robust estimates of dimensional stability through bootstrapping. Conceptually, it supports a dimensional perspective on psychopathology by capturing the complex interplay among symptoms.

In the present study, we apply EGA to a large sample of chronically ill patients with an acute exacerbation of schizophrenia at study baseline and endpoint. By doing so, we extend previous research beyond first-episode cohorts^9,10^ assessed only at baseline^11^. Chronically ill patients differ from first-episode samples, for example, in illness duration and cumulative treatment exposure. Thus, network structures identified in early-illness samples may not generalize to the chronic population, which constitutes the majority of patients in clinical practice. We examine bridge symptoms, defined as central items that connect different communities, to better understand how distinct symptom domains are interconnected. Assessing symptom networks at two time points allows us to explore whether community structures and bridge symptoms remain stable over the course of acute treatment or reorganize as symptoms improve. To our knowledge, only a single prior study has examined PANSS networks longitudinally in a comparable design^12^. Our study provides an independent replication in a different population (mainly Western vs. Chinese^12^) and extends the characterization of bridge symptoms beyond a single centrality metric.

We evaluate whether the community structures identified through EGA align with or diverge from widely accepted factor-analytic models of the PANSS, which allows us to situate network-based findings within the broader psychometric literature on the scale’s dimensional structure. In this way, we contribute to clarifying the applicability of network-based methods for modeling symptom structure in chronic schizophrenia.

## Methods

The available dataset is individual participant data (IPD) from a randomized controlled, double-blind trial by Tollefson et al.^14^. The trial compared the two antipsychotics haloperidol (*n* = 660) and olanzapine (*n* = 1336) in participants with schizophrenia over a period of six weeks. Detailed information on the study design and primary analyses can be found in the original publication.

The severity of psychiatric symptoms was measured using the PANSS. In our cross-sectional analyses, we include two measurement time points: baseline (week 0) and endpoint (week 6). To ensure comparability across both time points, only participants with observed values at the study endpoint were retained (*n* = 1213). We do not separate the sample by medication.

All analyses are conducted with the statistical software R^15^ version 4.4.1 (Windows 11). The package *EGAnet*^16^ is used to perform the explorative graph analysis (EGA) on the available dataset and 1000 resampled sets as part of a non-parametric bootstrapping approach. As network model estimation method, we apply the graphical least absolute shrinkage and selection operator (GLASSO) with Extended Bayesian Information criterion (EBIC) model selection. The result is a Gaussian Graphical Model (GGM) where nodes are partially correlated given all other nodes in the network^17^. The Walktrap algorithm detects the number and members of node communities without prespecified input. It performs random walks from any node with a distance of 3 steps to iteratively merge node pairs into groups. The existing edges determine the possible paths. The number of steps was chosen based on higher stability values of preliminary comparisons of different step distances. Ultimately, the algorithm aims to uncover the most meaningful clusters of symptoms or items. Based on the bootstrap results we can assess the stability of items and dimensions as proportion of times the empirical dimensions are exactly replicated. Values below a threshold of 0.70 are considered as instable^17^.

Further, we assess the role of nodes as central bridge symptoms. The GGM and its identified communities are provided to the bridge() function of the *networktools* package^18^ to evaluate the bridging role of individual nodes. As part of their respective communities, central bridge symptoms form numerous or strong connections with nodes from other communities.

To examine the robustness of the bridge centrality metrics, we compute the correlation stability (CS) coefficient using case-drop bootstrapping, as implemented in the *bootnet* package^19^. This procedure involves systematically removing cases to determine the stability of the rank order of central symptoms. Based on simulation studies, Epskamp et al. recommend a minimum CS value of 0.25 and a threshold of 0.5 for even greater interpretability^20^.

## Results

### Community detection

Table 1 presents the demographic and clinical characteristics of the sample at baseline. With a mean PANSS total score of 90, the sample is considered to be markedly ill^21^. We applied the community detection methods in 1000 non-parametric bootstrap iterations and display the frequency of dimensions found in Supplementary Table S1. The number of dimensions ranged from 3 to 7 at baseline and from 3 to 6 at endpoint, with the median being 5 for both measurement weeks. Five dimensions were identified in 85.9% of all cases at baseline and 76.3% of all cases at endpoint.

**Table 1:** Baseline characteristics.

	Total sample (*n* = 1213)
Age, years (mean, SD)	38.54 (11.57)
Sex, male (*n*, %)	781 (64.39)
Medication, olanzapine (n, %)	899 (74.11)
PANSS Total Score (mean, SD)	89.87 (18.93)

*PANSS* Positive and Negative Syndrome Scale.

**Fig. 1:**
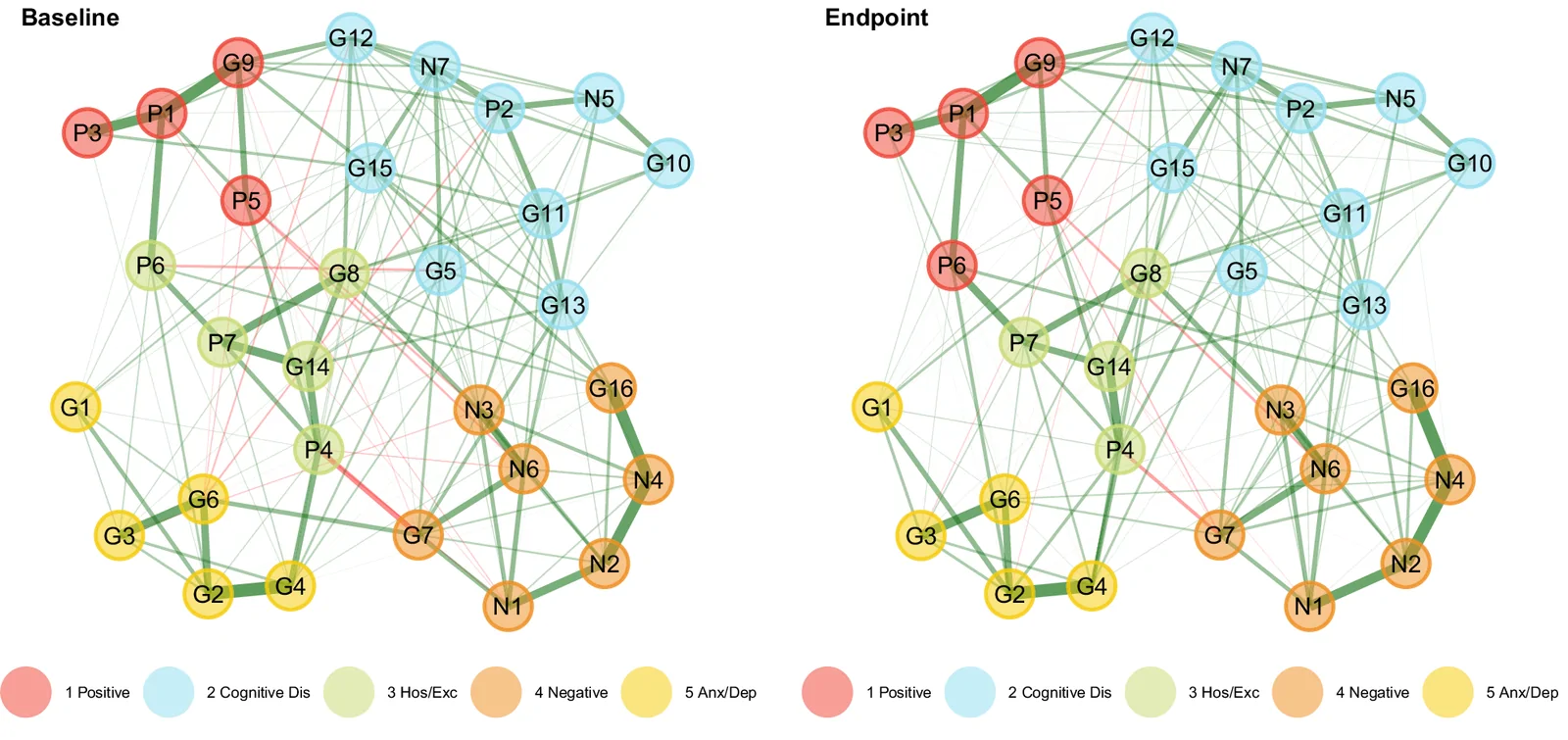
Explorative graph analysis. Left: baseline (week 0); Right: endpoint (week 6); Full sample of haloperidol & olanzapine; *n* = 1213 (observed at endpoint); Items: P1 (delusions), P2 (conceptual disorganization), P3 (hallucinatory behavior), P4 (excitement), P5 (grandiosity), P6 (suspiciousness), P7 (hostility), N1 (blunted affect), N2 (emotional withdrawal), N3 (poor rapport), N4 (passive social withdrawal), N5 (difficulty in abstract thinking), N6 (lack of spontaneity), N7 (stereotyped thinking), G1 (somatic concern), G2 (anxiety), G3 (guilt), G4 (tension), G5 (mannerisms and posturing), G6 (depression), G7 (motor retardation), G8 (uncooperativeness), G9 (unusual thought content), G10 (disorientation), G11 (poor attention), G12 (lack of judgment and insight), G13 (disturbance of volition), G14 (poor impulse control), G15 (preoccupation), G16 (active social avoidance).

Our factor solution is similar to the five factors identified previously^6–8^: positive symptoms, negative symptoms, hostility/excitement, anxiety/depression, and cognitive disorganization. The estimated networks at baseline and endpoint are shown in Fig. 1. To ensure comparability, we used a shared node layout for both networks, with the communities being numbered and identified by different colors.

### Community stability

Table 2 summarizes stability statistics in terms of structural consistency, as well as the average item stability of the identified communities. Structural consistency refers to the proportion of times the original dimension is exactly replicated across bootstrap samples. Among the identified communities in this network analysis, Community 3 (hostility/excitement) was the least stable, with a structural consistency of 0.267 at baseline and 0.635 at endpoint. Its average item stability values were reasonable (0.781 at baseline and 0.761 at endpoint). Communities 4 (negative symptoms) and 5 (anxiety/depression) proved to be the most stable groups. They showed the highest structural consistency (0.966 and 0.992 at baseline; 0.959 and 0.970 at endpoint) and the highest average item stability (0.988 and 0.998 at baseline; 0.985 and 0.993 at endpoint). By contrast, Community 1 (positive symptoms) shows a comparatively good structural consistency at baseline (0.763), and good item stability at baseline (0.930) and endpoint (0.857). Only at endpoint structural consistency was relatively low (0.531).

**Table 2:** Stability statistics of identified communities.

	Baseline	Endpoint
Structural consistency		
Community 1—Positive	0.763	0.531
Community 2—Cognitive Disorganization	0.715	0.712
Community 3—Hostility/Excitement	0.267	0.635
Community 4—Negative	0.966	0.959
Community 5—Anxiety/Depression	0.992	0.970
Average item stability		
Community 1—Positive	0.930	0.857
Community 2—Cognitive Disorganization	0.930	0.944
Community 3—Hostility/Excitement	0.781	0.761
Community 4—Negative	0.988	0.985
Community 5—Anxiety/Depression	0.998	0.993

Based on 1000 non-parametric bootstrap iterations; Structural consistency (proportion of times the original dimension is exactly replicated across bootstrap samples); Average item stability (per community).

### Composition of the communities

In Table 3 we present the items along with their labels, community assignments, and correspondence with three other important five-factor models of the PANSS. The Wallwork model and van der Gaag model have been identified previously as the best-fitting solutions in a systematic review^5–7^. The Marder model was included for comparison, as it is frequently used in pharmacological studies^5^.

**Table 3:** Item names and community assignments.

	Item	Name	Wallwork factor	Van der Gaag factor	Marder factor
Comm 1	P1	Delusions	Positive	Positive	Positive
Positive	P3	Hallucinatory behavior	Positive	Positive	Positive
	P5	Grandiosity	Positive	Positive	Positive
	G9	Unusual thought content	Positive	Positive	Positive
	P6*	Suspiciousness	-	Positive	Positive
Comm 2	P2	Conceptual disorganization	Cognitive	Cognitive	Cognitive
Cognitive	N5	Difficulty in abstract thinking	Cognitive	Cognitive	Cognitive
Disorg.	N7	Stereotyped thinking	-	Cognitive	Positive
	G5	Mannerisms and posturing	-	Cognitive	Cognitive
	G10	Disorientation	-	Cognitive	Cognitive
	G11	Poor attention	Cognitive	Cognitive	Cognitive
	G12	Lack of judgement and insight	-	Cognitive	Positive
	G13	Disturbance of volition	-	Cognitive	Cognitive
	**G15**	Preoccupation	-	Cognitive	Cognitive
Comm 3	**P4**	Excitement	Host/Exc	Host/Exc	Host/Exc
Hostility/	P6*	Suspiciousness	-	Positive	Positive
Excitement	P7	Hostility	Host/Exc	Host/Exc	Host/Exc
	**G8**	Uncooperativeness	Host/Exc	Host/Exc	Host/Exc
	G14	Poor impulse control	Host/Exc	Host/Exc	Host/Exc
Comm 4	N1	Blunted affect	Negative	Negative	Negative
Negative	N2	Emotional withdrawal	Negative	Negative	Negative
	N3	Poor rapport	Negative	Negative	Negative
	N4	Passive social withdrawal	Negative	Negative	Negative
	N6	Lack of spontaneity	Negative	Negative	Negative
	G7	Motor retardation	Negative	Negative	Negative
	G16	Active social avoidance	-	Negative	Negative
Comm 5	G1	Somatic concern	-	Anx/Dep	-
Anxiety/	G2	Anxiety	Anx/Dep	Anx/Dep	Anx/Dep
Depression	G3	Guilt	Anx/Dep	Anx/Dep	Anx/Dep
	G4	Tension	-	Anx/Dep	Anx/Dep
	G6	Depression	Anx/Dep	Anx/Dep	Anx/Dep

* Item P6 (suspiciousness) is listed in two communities, as it was assigned to Community 3 (hostility/excitement) at baseline and to Community 1 (positive symptoms) at endpoint. Items in bold are considered as bridge symptoms.

All items of the Wallwork and van der Gaag solutions fit exactly to the five communities identified by network analysis at study endpoint (Table 3). Of note, the factor analyses, which had led to the Wallwork factors, dropped 10 of the 30 PANSS items. Therefore, our five communities have more items than those of Wallwork et al. For example, our solution for the cognitive disorganization community has nine items and the corresponding Wallwork factor includes only three items.

Moreover, item P6 (suspiciousness) is dropped in the Wallwork model but is part of Community 3 (hostility/excitement) at baseline and switches to Community 1 (positive symptoms) at endpoint in our solution. It is therefore discrepant at baseline to the assignments of the van der Gaag model and the Marder model. Only the Marder model had additional discrepancies in Community 2 (cognitive disorganization) where Marder et al. considers the items N7 (stereotyped thinking) and G12 (lack of judgement and insight) to be positive symptoms. Therefore, most variability was found in the cognitive disorganization PANSS factor (Community 2).

### Stability of item assignments

**Fig. 2:**
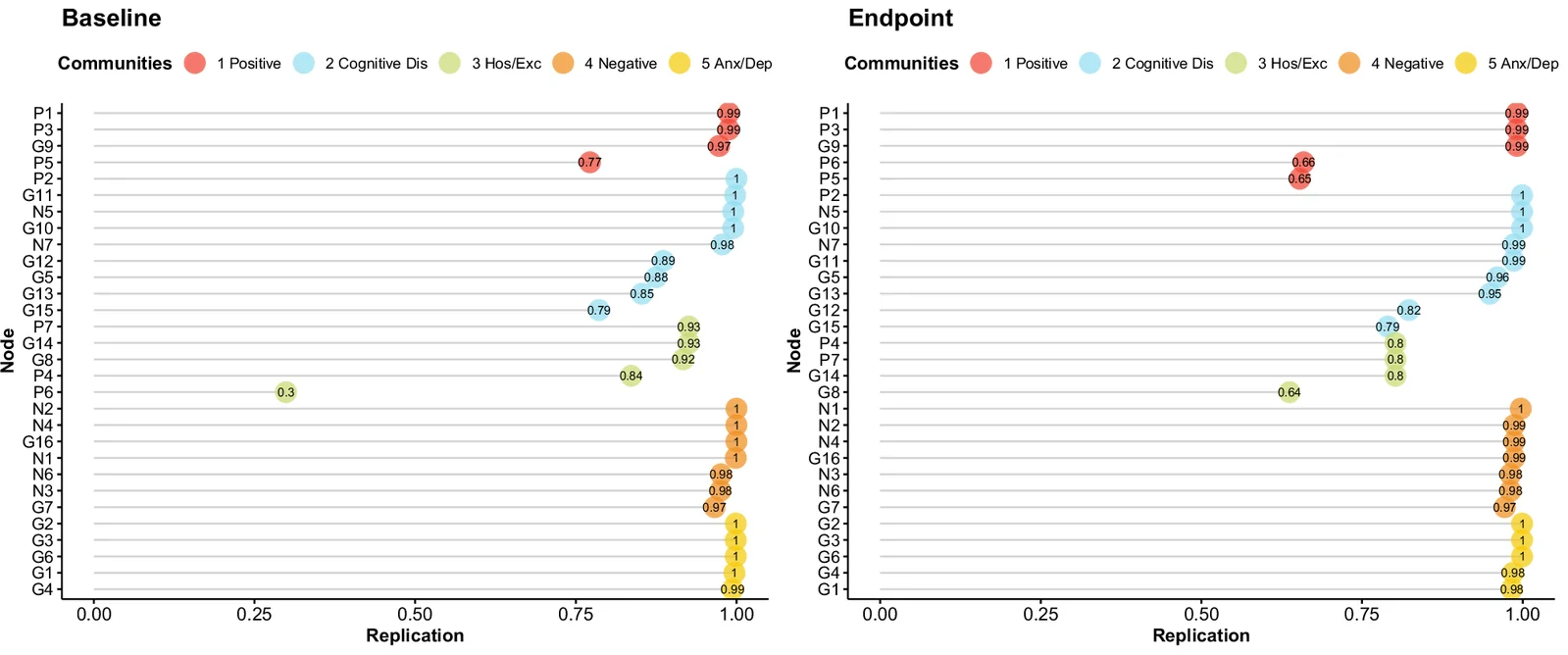
BootEGA results. Item stability in 1000 bootstrap replications.

Figure 2 displays the stability of individual items across 1000 bootstrap replications. At baseline, only item P6 (suspiciousness) falls below the recommended threshold of 0.7, with a value of 0.3. As part of Community 3 (hostility/excitement), it lowers the average stability of the entire community (see Table 2). All other items show replication proportions between 0.77 and 1.0 and can therefore be considered stable in their assignments. At study endpoint, items P5 (grandiosity, 0.65), P6 (suspiciousness, 0.66), and G8 (uncooperativeness 0.64) fall below the threshold, thereby also reducing the stability metrics of Communities 1 (positive symptoms) and 3 (hostility/excitement).

### Loadings of unstable items

Table 4 shows the proportions with which the previously identified (Fig. 2) unstable items were assigned to dimensions across the 1000 replications. In other words, we explore how often the unstable items load on different communities. At baseline, item P6 (suspiciousness) was most frequently assigned to Dimension 1 (positive symptoms, 0.587) but was also allocated to Dimension 3 (hostility/excitement, 0.299). We can see a similar pattern at endpoint (D1: 0.659, D3: 0.244). This explains the possibility of the community switch observed in Fig. 1 even though bootstrap replications favor a consistent assignment to Dimension 1 (positive symptoms) across both time points. Item P5 (grandiosity) was most often assigned to Dimension 1 (positive symptoms, 0.653), followed by Dimension 2 (cognitive disorganization, 0.206) and Dimension 3 (hostility/excitement, 0.122). Item G8 (uncooperativeness), in turn, was most frequently placed in Dimension 3 (hostility/excitement, 0.637), but also showed a notable allocation to Dimension 2 (cognitive disorganization, 0.317). These values support the possibility of multidimensional items.

**Table 4:** Replication frequency of instable items by measurement point.

PANSS items	D1	D2	D3	D4	D5	D6	D7
Baseline							
P6 suspiciousness	0.587	0.060	0.299	0.000	0.024	0.029	0.000
Endpoint							
P5 grandiosity	0.653	0.206	0.122	0.000	0.003	0.016	-
P6 suspiciousness	0.659	0.012	0.224	0.000	0.091	0.014	-
G8 uncooperativeness	0.005	0.317	0.637	0.000	0.038	0.003	-

Based on 1000 non-parametric bootstrap iterations; Separated by measurement points; Replication frequency (proportion of bootstraps that a variable appeared in each dimension); PANSS (Positive and Negative Syndrome Scale): P5 (grandiosity), P6 (suspiciousness), G8 (uncooperativeness).

### Bridge symptoms

Bridge centrality metrics assess a node’s role in linking different item communities. Bridge strength is defined as the sum of absolute edge weights connecting a node to nodes outside its own community, while bridge expected influence also accounts for the sign of edges. Thus, in networks where all edges are positive, strength and expected influence are equivalent. Bridge closeness reflects how close a node is to other communities, based on shortest path lengths calculated as the inverse of absolute edge weights. Bridge betweenness quantifies how often a node lies on shortest paths between communities, highlighting key intermediaries. For closeness and betweenness, negative edges are omitted^18,22^.

**Fig. 3:**
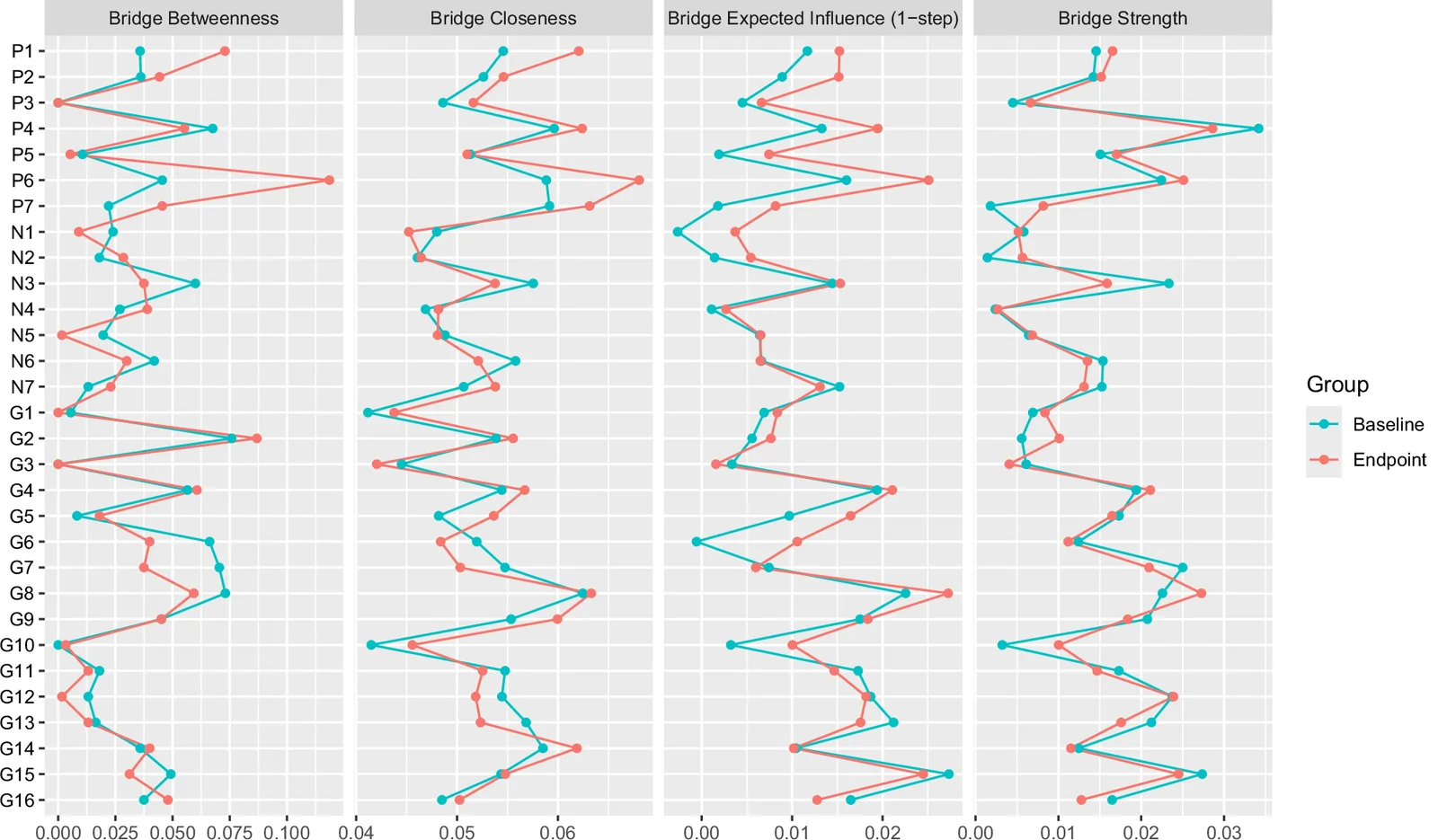
Bridge metrics. Normalized bridge centrality metrics.

For the empirical networks at baseline and endpoint, stable centrality and bridge centrality metrics were obtained from 1000 case-drop bootstraps (Supplementary Fig. S1). CS coefficients ranged from 0.517 (e.g., bridge betweenness at both baseline and endpoint) to 0.750 (bridge strength at baseline). All metrics are well above the minimum threshold of 0.25 and also exceed the more conservative threshold of 0.5. The bridge centrality results shown in Fig. 3 can therefore be interpreted as stable. To account for differences in community size, the metrics were normalized for comparison.

Figure 3 compares the four bridge metrics of the network nodes at baseline and endpoint. Within Community 3 (hostility/excitement), items P4 (excitement) and G8 (uncooperativeness) show high values, i.e. strong bridge characteristics, across all metrics and both time points. Item P6 (suspiciousness) ranks among the top three for all metrics only at endpoint, when it is assigned to Community 1 (positive symptoms). This is because it still has strong connections with the items of Community 3 (hostility/excitement), especially to item P7 (hostility). In addition, item G15 (preoccupation) from Community 2 (cognitive disorganization) shows elevated values only for bridge strength and bridge expected influence.

## Discussion

Most analyses of the PANSS structure to date have relied on classical factor analysis. It is important to further validate these findings with different methodological approaches. In a large sample of 1213 patients with schizophrenia, we provide additional evidence for the presence of a five-factor model with EGA and non-parametric bootstraps. Factor solutions with fewer or more dimensions were rarely observed across 1000 iterations. Interestingly, the main factor models previously identified through factor analysis, such as those proposed by Wallwork, van der Gaag, and Marder, align with our findings in terms of both the number of factors and item assignments. Accordingly, their commonly used labels can be adopted here as well. This replication is particularly important, as there are already independent scales that adopt these factors (e.g., Brief Evaluation of Psychosis Symptom Domains (BE-PSD))^23^. Moreover, this type of analysis provided additional perspectives on item and community stability, replication frequency, and bridge symptoms which is not possible by using factor analysis.

Our discussion focuses on two related findings: item instability and the presence of key bridge symptoms connecting different PANSS dimensions. Three items did not meet the recommended stability threshold of 0.7, also affecting the consistency of their assigned communities. Notably, in the Wallwork model, which was identified in a recent systematic review as one of the most promising factor models^5^, unstable item P6 (suspiciousness) was excluded from the final structure due to inconsistent assignments. In our data, P6 (suspiciousness) was most frequently assigned to Community 1 (positive symptoms) at both time points (see Table 4), as reflected in our endpoint network, but it was also partially assigned to Community 3 (hostility/excitement), as seen in the baseline network. Despite some variation, it still corresponds predominantly to the positive symptom community. Similarly, the unstable items P5 (grandiosity) and G8 (uncooperativeness) were ultimately and most consistently assigned to the same communities as in previous factor models and replicate the van der Gaag model exactly. In general, these three items could be considered candidates for exclusion, as was done in the Wallwork model. However, the Wallwork model excludes 10 of the 30 PANSS items, some of which are clinically relevant especially in terms of the cognitive disorganization domain which only includes three items.

In previous research, the cognitive disorganization factor has been identified as the least robust among the five PANSS factors in terms of item assignment^5^ and is therefore substantially reduced in the Wallwork model. In contrast, our stability analyses indicate that the Hostility/Excitement community shows more structural instability. Stability depends on strong within-community and weaker between-community connections. The identification of central bridge symptoms helps to identify specific items that contribute to instability by forming strong connections between communities.

In this regard, Community 2 (cognitive disorganization) contains the central bridge symptom G15 (preoccupation), and Community 3 (hostility/excitement) includes P4 (excitement) and G8 (uncooperativeness). All three symptoms exhibit strong or frequent connections with other communities, which is visually apparent in Fig. 1, where they are centrally positioned within the network. At endpoint, P6 (suspiciousness) is part of Community 1 (positive symptoms) but remains strongly connected to Community 3 (hostility/excitement), also reflecting its multidimensionality. This multidimensionality may be driven by the conceptual heterogeneity found in the original PANSS definition of P6. Severity ratings of 4 or below denote generalized distrust without persecutory delusions, positioning the item conceptually near the hostility/excitement domain, whereas ratings of 5 and above explicitly require clear persecutory delusions, aligning P6 more closely with Community 1 (positive symptoms). A similar pattern applies to G15, whose anchors shift from non-specific self-absorption at lower ratings to autistic preoccupation with responses to possible hallucinations at ratings of 5 and above.

The apparent switch in P6’s community membership between baseline and endpoint reflects a difference in how decisively each sample-specific network resolved P6’s inherent multidimensionality. At endpoint, bootstrap replications clearly favored Community 1 (65.9% vs. 22.4%, Table 4). At baseline, Community 1 was also the more frequent outcome (58.7% vs. 29.9%), but the margin was smaller. Therefore, the decision for our data sample is a minority outcome. Together with the existence of the other bridge symptoms, Community 3 contains most multi-dimensional items, and therefore is less stable than the other communities.

The pronounced structural inconsistency of Community 3 at baseline (0.267) illustrates the difference between item-level and community-level stability. Its average item stability (0.781) indicates that, individually, items were still reliably assigned to Community 3 across replications. However, its structural consistency was low, meaning the community as an exact set of items was rarely replicated in identical form. Even when the remaining items stay reliably grouped together, a single boundary item that intermittently changes communities is sufficient to prevent exact replication of the community. Community 3’s bridge items (P4, P6, and G8) allow neighboring items to occasionally be pulled toward or away from Community 3 across resamples. Community 1 showed an analog pattern with items P5 and P6 at endpoint.

Taken together, the instability of communities 1 and 3 across time points is due to a few recurring boundary items (P5, P6, and G8), rather than a broad reorganization of the identified dimensional structure.

There is only limited research applying EGA to the PANSS, and the existing studies have primarily focused on first-episode or Asian subgroups and baseline data. Recently, Cheng et al. identified five different communities in a sample of 147 first-episode Chinese patients in a study on cognition, where PANSS items were combined with subscales from the Wechsler Adult Intelligence Scale^9^. These communities include cognitive function, predominantly positive symptoms, predominantly negative symptoms, excitement/grandiosity, and affect-related symptoms. While the overarching categories are similar to ours, there are notable differences in the specific item assignments. The authors even excluded six PANSS items that did not exhibit any connections to other nodes in the network.

Dal Santo et al. analyzed baseline data from 446 European first-episode patients with minimal or no prior antipsychotic treatment^10^. Their results grouped 21 PANSS items into four communities: positive, cognitive/disorganized, excited/aggressive, and negative. For their final solution, items previously identified as unstable were omitted, resulting in a reduced item pool. The item assignments correspond to ours, except that their solution completely removes the anxiety/depression community.

Comparable to our results, Serafim et al. found five communities comprising positive symptoms, negative symptoms, as well as a cognitive/disorganization domain, affective/depression-anxiety domain, and hostility/excitement domain in a Brazilian sample of 1262 participants^11^. Item assignments show only four discrepancies to our solution for items P6 (suspiciousness), G7 (motor retardation), G12 (lack of judgement and insight), and G15 (preoccupation). The reported instability of these items and corresponding replication frequency across different communities suggest additional proximity to our solution.

Finally, Sun et al. identified five PANSS communities in a sample of 3030 Chinese participants treated with seven different antipsychotics over a period of six weeks^12^. Their study offers the most comparable framework to ours, as they also analyzed networks at baseline and endpoint without excluding PANSS items or supplementing them with additional scales, and identified bridge symptoms using bridge strength. The resulting communities closely matched ours in both labeling and item assignment, with discrepancies observed in five items: P5 (grandiosity), G5 (mannerisms and posturing), G7 (motor retardation), G12 (lack of judgment and insight), and G16 (active social avoidance). Instead of the term *Cognitive Disorganization*, they used *Disorganized Thought* to describe Community 2. The authors identified P2 (conceptual disorganization), P4 (excitement), P7 (hostility), G8 (uncooperativeness), and N5 (difficulty in abstract thinking) as bridge symptoms, of which P4 and G8 overlap with our findings.

Our study is subject to certain limitations. The analyses relied solely on observed cases, which may limit the generalizability of the findings due to a high dropout rate. However, this approach allowed for direct comparisons across time points without relying on data imputation and its associated statistical assumptions. Moreover, although the PANSS is a well-established instrument for assessing schizophrenia symptoms, it does not capture the full spectrum of psychopathology, such as sleep disturbances or compulsive symptoms, and it is not a diagnostic tool. Future studies could benefit from incorporating complementary measures or forming a new scale to represent a broader range of symptom domains, thereby supporting the growing shift toward a dimensional rather than categorical understanding of psychiatric disorders.

## Supplementary information

Supplemental Material

Analysis Code

## Data Availability

The R code used to conduct the exploratory graph analysis reported in this study, together with an example dataset and full session information (R version, RStudio version, and package versions), is provided as Supplementary Information.
