# Supplementary material for: Revisiting symptom dimensions in schizophrenia with exploratory graph analysis
Source: Schizophrenia (Heidelb). 2026 Sep 7;12(1):72. doi: 10.1038/s41537-026-00799-y (PMC13550554; doi:10.1038/s41537-026-00799-y)
Supplement: Supplementary file 1 [file 41537_2026_799_MOESM1_ESM.docx]

**Revisiting Symptom Dimensions in Schizophrenia with Exploratory Graph Analysis**

Supplementary Material

# Supplementary Figures

Figure S1: Correlation stability coefficients (bootnet)

# Supplementary Tables

Table S1: Frequency of Dimensions

|  | **Baseline** | **Endpoint** |
| --- | --- | --- |
| 3 Dimensions | 0.001 | 0.006 |
| 4 Dimensions | 0.073 | 0.191 |
| 5 Dimensions | 0.859 | 0.763 |
| 6 Dimensions | 0.064 | 0.040 |
| 7 Dimensions | 0.003 | - |
| Median Dimensions [95%CI] | 5 [4.23, 5.77] | 5 [4.06, 5.94] |

*Note*: Based on 1000 non-parametric bootstrap iterations; Frequency (proportion of times the number of dimensions was identified)
